# Hepatopancreas Transcriptome Analysis of *Spinibarbus sinensis* to Reveal Different Growth-Related Genes

**DOI:** 10.3390/genes15070949

**Published:** 2024-07-19

**Authors:** Bo Zhou, Leyan Ling, Bin Wang, Fei Yang, Mengdan Hou, Fan Liu, Yu Li, Hui Luo, Wenping He, Hua Ye

**Affiliations:** 1 Fisheries Institute, Sichuan Academy of Agricultural Sciences, Yibin 644000, China; zhoubo2008@163.com (B.Z.); wangbinhsimple@163.com (B.W.); yangfei170140@163.com (F.Y.); 2 Key Laboratory of Freshwater Fish Reproduction and Development (Ministry of Education), Key Laboratory of Aquatic Science of Chongqing, College of Fisheries, Southwest University, Chongqing 402460, China; lly12022024@163.com (L.L.); hou660x@163.com (M.H.); l0212f@163.com (F.L.); ly085020@126.com (Y.L.); luohui2629@126.com (H.L.); hewenping2008@163.com (W.H.)

**Keywords:** *Spinibarbus sinensis*, hepatopancreas transcriptome, growth, energy metabolism, neuroendocrine regulation

## Abstract

*Spinibarbus sinensis*, also known as Qingbo, is an important economic fish in China. However, the detailed mechanisms underlying its growth are still unknown. To excavate the genes and signaling pathways related to its growth, we compared the transcriptome profiles of the hepatopancreas tissues of *S. sinensis*, with two groups of growth rate for evaluation. An average of 66,304,909 and 68,739,585 clean reads were obtained in the fast growth (FG) and slow growth (SG) group, respectively. The differential gene expression analysis results showed that 272 differentially expressed genes (DEGs) were screened between the FG and SG groups, including 101 up-regulated genes and 171 down-regulated genes. Gene Ontology (GO) and Kyoto Encyclopedia of Genes and Genomes (KEGG) enrichment analysis results showed that GO terms related to metabolic process, organic substance metabolic process, and catalytic activity were enriched, pathway signals related to steroid biosynthesis and protein digestion and absorption were also detected. Meanwhile, the potential key regulatory genes *sst2*, *fndc4*, and *cckra* related to the growth of *S. sinensis* were screened. Reverse transcript fluorescence quantitative PCR (RT-qPCR) validation of 18 DEGs associated with growth differences showed that the RT-qPCR results were consistent with RNA-seq analysis, and nine genes, *stk31*, *gpr149*, *angptl1*, *fstl1*, *sik1*, *ror2*, *nlrc3*, *pdlim2*, and *nav2* were significantly expressed in the FG group. *bmp1*, *stc1*, *gpatch8*, *sstrt2*, *s100a1*, *ktf6*, *cckar6*, *sync1*, *bhlha15*, a total of nine genes were significantly expressed in the SG group. This study provides basic information for improving the growth characteristics of *S. sinensis* and the functional research of candidate genes.

## 1. Introduction

Qingbo (*S*. *sinensis*), belonging to *Spinibarbus*, is an important and economic fish in China. It is mainly distributed in the main and tributary streams of the middle and upper reaches of the Yangtze River [1]. Males and females sexually mature at 3–4 years old and 4–5 years old, respectively. *S. sinensis* is an omnivorous fish and its diet includes higher plants, conglomerate algae, aquatic insects, and *Limnoperna fortune*, and grows rapidly before the age of 4 years old and slower after the age of 5 years old. The body weight of *S. sinensis* at 2 years old can reach 400 g under artificial breeding conditions, and the market specifications are generally 500–1000 g [1,2]. *S. sinensis* is one of the most popular species on the Chinese table as well as in the aquaculture industry [3]. However, in recent years, the wild resources of *S. sinensis* have declined due to ecological and environmental destruction, overfishing, and increasing water pollution. Therefore, it is necessary to conduct research related to the growth traits of *S. sinensis*. Speeding its growth rate could not only shorten its culture cycle to save farming costs but also increase its production, providing economic benefits and meeting the high demand for *S. sinensis* in the market. However, current research on *S. sinensis* mainly focuses on stress physiology [4,5,6], biological characteristics [1,7,8], and disease resistance and immunity [9,10], with relatively few reports on genetic studies of its growth. The foundation research for its large-scale selection breeding with improved traits is also relatively weak.

With the development of high-throughput sequencing, there are lots of studies focusing on the exploration of candidate genes and regulatory pathways related to fish growth based on RNA-seq. Li et al. [11] conducted transcriptome analysis on the muscle of *Cyprinus carpio* with different body sizes at 20 months old, identifying nine functional genes related to the ubiquitin–proteasome pathway and several genes related to muscle contraction. Zhang et al. [12] used RNA-seq to analyze the muscle and liver tissues of the same batch of *Mylopharyngodon piceus*. Through GO and KEGG enrichment analysis, they found significant differences in the expression of genes related to growth and development-associated metabolic pathways. Liu et al. [13] performed transcriptome analysis on the muscles of 3-month-old *Siniperca chuatsi* with different body sizes and explored its specific growth-related differentially expressed genes (DEGs).

The hepatopancreas functions as a nutrition sensor in teleost fish, assisting in the storage of nutrients and energy, controlling nutritional status, and regulating physiological metabolism [14]. It contains a lot of energy-supplying substances that can provide energy, particularly lipids, which can be used by aquatic animals for energy-intensive processes like growth, molting, gonad development, etc. [15,16]. In this study, we performed RNA-seq of hepatopancreas tissues of *S. sinensis* with different growth rates and obtained databases of their hepatopancreas transcripts. Then, we performed gene function annotation and expression profile analysis. To identify DEGs associated with their different growth rates, we also further selected 18 candidate genes by sequencing analysis and validated them by RT-qPCR. These findings will provide basic information for improving the growth traits of *S. sinensis* and contribute to further functional studies of genes related to growth.

## 2. Materials and Methods

### 2.1. Ethics Statement, Experimental Animals, and Sample Collection

All operations of this study were performed in compliance with the Animal Management Regulations of the Animal Welfare and Ethical Committee of Southwest University (Chongqing, China) and the Use Committee of Fisheries Research Institute, Sichuan Academy of Agricultural Sciences (20200630001A, 20200630002A). The same batch of artificially bred 31-month-old F1-generation (the parents were 8 males and 20 females) *S. sinensis* was selected in this study, which came from the Yibin Base of the Fisheries Research Institute of the Sichuan Academy of Agricultural Sciences. They were fed twice a day (9:00 a.m. and 6:00 p.m.) under the same pool with commercial food at 3% of the body weight. After weighing, the individuals of the top 10% and bottom 10% of body weight were selected as the fast growth (FG) group and slow growth (SG) group, respectively.

### 2.2. Date Measurement and Sample Collection

The body weight and full length of *S. sinensis* from the FG and SG groups were measured. Then, a total of 9 individuals (3 replications and 3 fish/replication) from the FG group and 9 individuals (3 replications and 3 fish/replication) from the SG group were randomly selected for sampling. Before sample collection, MS-222 (100 mg/L) was used to anesthetize experimental fish. The hepatopancreas tissues of the fish were rapidly isolated and kept in RNA protect solution (Takara Biotechnology, Tianjin, China) overnight, then frozen at −80 °C for RNA extraction.

### 2.3. RNA Isolation, Transcriptome Library Construction, and Sequencing

A Trizol kit (Invitrogen, Carlsbad, CA, USA) was used to extract the total RNA from the hepatopancreas tissues. A Qubit 2.0 Fluorometer was used to determine the concentration of the RNA after the contaminating genomic DNA was removed using DNase I (Takara Biotechnology, Tianjin, China). After that, an Agilent 2100 Bioanalyzer was used to confirm the RNA integrity. Next, according to our previous protocol [17], the first and second strands of cDNA were synthesized and purified. The cDNA was sequenced using an Illumina NovaSeq 6000 PE 150 at Novogene Technology Co., Ltd. (Tianjin, China).

### 2.4. Data Processing, Assembly, and Functional Annotation

First, the raw reads were processed using Trimmomatic V0.39 to ensure high analytical quality for the RNA sequencing. Reads were filtered out if they met the following criteria: (1) more than 50% low-quality bases (Q-value ≤ 20) [18]; (2) contained adapters or more than 10% unknown nucleotides (N) [19]. After filtering, Trinity V2.2.1 was employed to assemble the clean reads de novo. The longest transcript from each cluster was selected as the unigene sequence for hierarchical clustering analysis using Corset V1.09 [20]. Gene annotations were conducted using the Nr, Nt, KOG, Swiss-Prot, and Uniprot databases via online BLAST tools, the National Center for Biotechnology Information (NCBI) (https://www.ncbi.nlm.nih.gov/tools/primer-blast/index.cgi) (accessed on 10 February 2021), Diamond V0.9.14.115, and BLASTn V2.11.0+, providing comprehensive gene function information.

### 2.5. Gene Ontology (GO) and Kyoto Encyclopedia of Genes and Genomes (KEGG) Enrichment Analysis of DEGs

Differential expression analysis of RNA was conducted for the FG and SG groups using the DESeq2 R package (1.20.0). DEGs were identified with the criteria of |log2(Fold Change)| > 1 and an FDR value (padj) < 0.05. To understand the functions of DEGs in hepatopancreas tissues, Gene Ontology (GO) and Kyoto Encyclopedia of Genes and Genomes (KEGG) enrichment analysis of DEGs was performed using the cluster Profiler (3.8.1) software.

### 2.6. Validation of the RNA-seq Analysis by RT-qPCR

A total of 18 DEGs (9 up-regulated and 9 down-regulated DEGs, respectively) from the transcriptome data were randomly selected for RT-qPCR validation. Primer sequences for these genes are listed in Table 1. Reverse transcription was performed using a PrimeScript™ RT Reagent Kit with gDNA Eraser (Takara Biotechnology, Tianjin, China) to synthesize cDNA. The qRT-PCR was conducted on an ABI QuantStudio 3 Real-Time PCR System (Thermo Fisher Scientific, Shanghai, China) in a total reaction volume of 10 μL. The reaction mixture included 5 μL of 2 × TB Green Premix Ex Taq II (Takara Biotechnology, Tianjin, China), 0.2 μL of ROX Reference Dye II (50×), 3 μL of ddH_2_O, 1 μL of template cDNA, and 0.4 μL of each primer (10 μM). β-actin was used as the internal reference gene, and relative gene expression levels were calculated using the 2^−ΔΔCT^ method.

### 2.7. Statistical Analysis

Statistical analysis of the results was performed using SPSS Statistics 27.0 software. All data were subjected to independent *t*-tests and significant differences were considered when *p* < 0.05.

## 3. Results

### 3.1. Analysis of Growth Data between FG and SG

**Figure 1:**
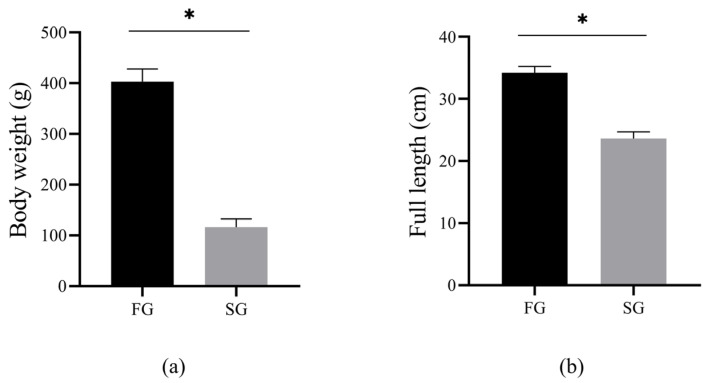
The statistics of body weight (**a**) and full length (**b**) between FG and SG (* *p* < 0.05).

The body weight and full length of the FG and SG groups are shown in (Figure 1a,b). Briefly, the body weight (FG: 403.25 ± 24.92 g; SG: 115.91 ± 16.23 g) and full length (FG: 34.23 ± 1.02 cm; SG: 23.60 ± 1.13 cm) of the FG group were significantly higher than those of the SG group (*p* < 0.05).

### 3.2. Preliminary Analysis of the Transcriptome Sequences

**Figure 2:**
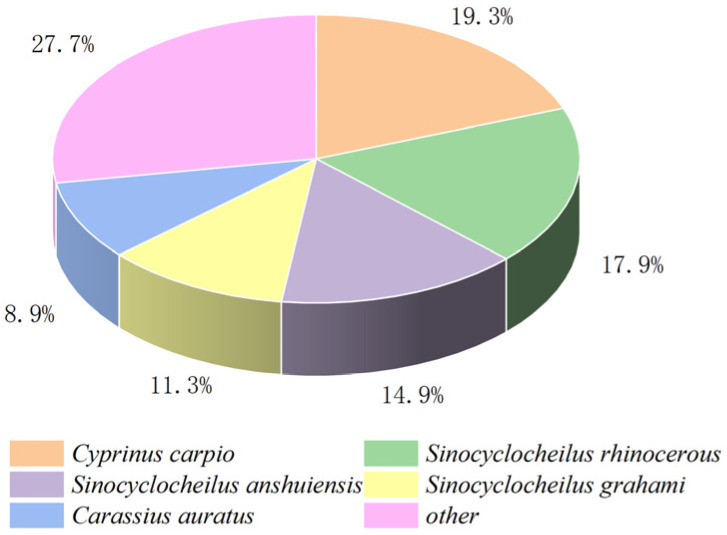
The alignment map of Nr library species.

We performed a comparative transcriptome analysis of the hepatopancreas tissues between the FG and SG groups. The analysis yielded an average of 67,049,644 and 69,445,985 raw reads for the FG group and SG group, respectively. An average of 66,304,909 and 68,739,585 clean reads and a total of 40.51 Gb clean bases were also obtained. The GC content ranged from 45.79% to 47.18% of the sequencing data with Q30 > 93% set as a filter (Table 2), indicating that the data used for subsequent analysis were high-quality. Then, a total of 150,030 unigenes were generated with an average length and N50 length of 1050, and 1635 bp, respectively. All the raw data obtained in this study have been submitted to the NCBI Sequence Read Archive (SRA) database under the project (PRJNA1109805). A total of 52,662 (35.10%), 144,091 (31.83%), 25,498 (16.99%), 36,740 (24.48%), 39,583 (26.38%), 39,577 (26.37%), and 13,553 (9.03%) genes were matched with the NCBI-Nr, NCBI-Nt, KO, SwissProt, PFAM, GO, and KOG databases. According to the annotation results of the Nr database, *S. sinensis* shared the highest identity with *C. carpio* (19.3%), *Sinocyclocheilus rhinocerous* (17.9%), *S. anshuiensi* (14.9%), *S. grahami* (11.3%), and *Carassius auratus* (8.9%) (Figure 2).

### 3.3. DEGs Analysis

**Figure 3:**
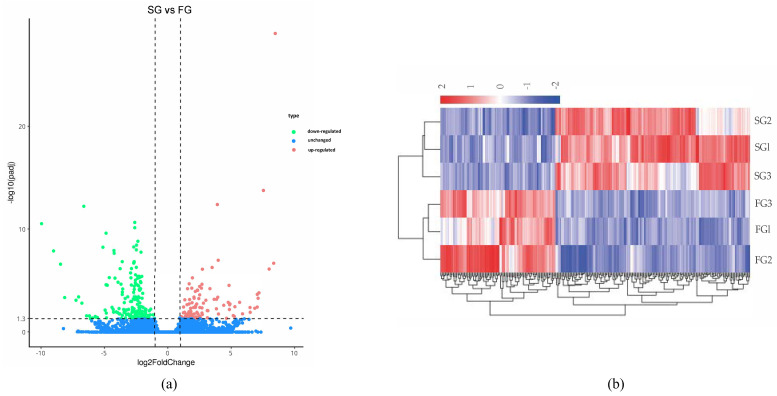
Overview of differentially expressed genes of *S. sinensis*. (**a**) Volcano plot of transcriptome differentially expressed genes; (**b**) heat map of differentially expressed genes.

We screened the high-confidence DEGs expressed in the hepatopancreas of *S. sinensis* with different growth rates with a standard of |log2(Fold Change)| > 1 and *p* < 0.05. The results indicated that 272 DEGs were found between the FG and SG groups. A total of 101 DEGs were up-regulated and 171 were down-regulated (Figure 3a). A further hierarchical cluster analysis showed that the expression patterns were quite different between the pairwise comparison groups, while similar among different varieties in the group (Figure 3b).

### 3.4. Enrichment and Pathway Analysis of GO and KEGG

**Figure 4:**
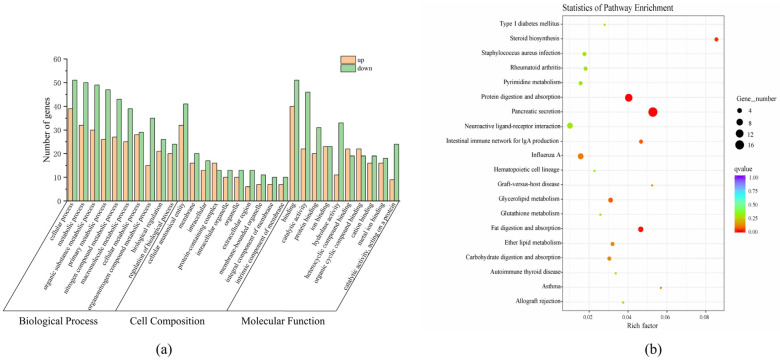
Analysis of differentially expressed genes. (**a**) Gene ontology assignment of differentially expressed genes of *S. sinensis*; (**b**) KEGG pathway of differentially expressed genes of *S. sinensis*.

A total of 1156 GO functional annotations were obtained from the 272 DEGs in the GO functional classification system. Among them, 727 were Biological Processes (BPs), 289 were Molecular Functions (MFs), and 139 were Cell Components (CCs). Based on the number of enriched DEGs, the top 10 enriched GO terms for each category were plotted in a histogram (Figure 4a). In the BP category, the top three enriched terms were cellular process (GO:0009987), metabolic process (GO:0008152), and organic substance metabolic process (GO:0071704). For the CC category, cellular anatomical entity (GO:0110165), membrane (GO:0016020), and intracellular (GO:0005622) were the most enriched terms. In the MF category, the enriched DEGs were primarily associated with binding activity (GO:0005488), catalytic activity (GO:0003824), and protein binding (GO:0005515). Additionally, some DEGs were also enriched in GO terms related to the primary metabolic process (GO:0044238), nitrogen compound metabolic process (GO:0006807), and macromolecule metabolic process (GO:0043170).

In addition, a total of 121 specific metabolic pathways were enriched in this study. Among them, 12 pathways were significantly enriched (*p* < 0.05). The most significantly enriched KEGG pathways included pancreatic secretion (ko04972), protein digestion and absorption (ko04974), steroid biosynthesis (ko00100), and fat digestion and absorption (ko04975) (Figure 4b). Additionally, other lipid metabolism-related pathways, such as glycerolipid metabolism (ko00561) and ether lipid metabolism (ko00565), were also identified through KEGG enrichment analysis.

### 3.5. Verification of DEGs with qRT-PCR

**Figure 5:**
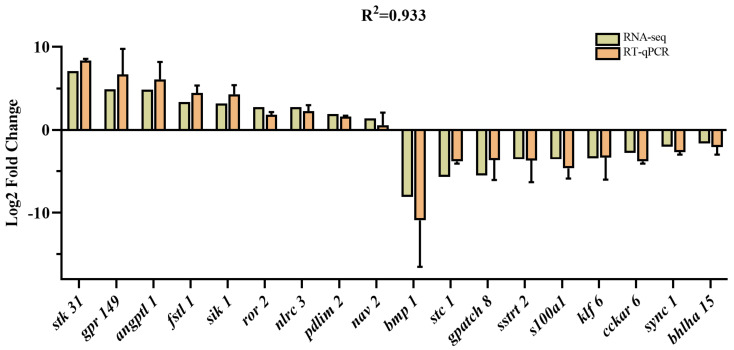
Comparison of the expression of DEGs between RNA-seq and qRT-PCR.

We selected 18 DEGs based on our RNA-seq analysis and previous studies for RT-qPCR validation [15,21,22]. The results showed that the expression level of *stk 31*, *grp 149*, *angptl 1*, *fstl 1*, *sik 1*, *ror 2*, *nlrc 3*, *pdlim 2*, and *nav 2* were highly expressed in the FG group, while the expression level of *bmp*, stc 1, *gpatch 8*, *sst 2*, *s100a1*, *klf 6*, *cckar*, *sync 1*, and *bhlha 15* were lowly expressed. The gene expression patterns detected via qRT-PCR were strongly correlated with the transcriptome sequencing data (Figure 5). Overall, the results of the qRT-PCR were consistent with the transcriptome analysis and supported the reliability of the transcriptome data.

## 4. Discussion

The growth of fish is not only related to the external environment such as feeding, temperature, and water quality but is also affected by internal factors such as differences in the capacity of metabolic organs between individuals [23,24]. In teleost fish, there are several organs involved in feeding and energy metabolism. Among them, the hepatopancreas has been recognized as one of the largest and most important organs, which plays a key role in carbohydrate and lipid metabolism, nutritional status, and energy storage and decomposition [14,25,26]. To investigate the molecular mechanisms of different growth rates of *S. sinensis*, here, we constructed six hepatopancreas cDNA libraries based on RNA-seq. In total, we obtained 150,030 unigenes from 69,445,985, and 67,049,644 raw reads, facilitating the perfection of teleost fish gene expression profiles.

The growth of fish relies on the metabolism and transformation of various nutrients within their bodies, and one of the important ways for fish to obtain nutrients is through feeding [27,28]. In this experiment, *cckra* was highly expressed in the SG group. Cholecystokinin (*cck*) was considered a satiety signal that inhibits food intake by slowing down gastrointestinal motility and reducing the secretion of digestive fluids, thus suppressing the ingestion of feeds [29,30]. *cckra* is the active receptor of *cck*. Hence, the activation of *cck* by *cckra* might inhibit the feeding activity of *S. sinensis*, leading to insufficient nutrient intake, and slower growth and development. Notably, somatostatin 2 (*sst 2*) was identified as highly expressed in the SG group. Somatostatin (*sst*) inhibits the secretion of growth hormone (GH) by the pituitary, and affects glycolipid metabolism by inhibiting the secretion of insulin and glucagon. At the same time, *sst* regulates fish feeding by inhibiting the secretion of gastrointestinal peptides such as *cck* [31,32]. Therefore, we hypothesized that *sst2* affects the growth of *S. sinensis* by inhibiting the secretion of GH and *cck*.

Lipids and carbohydrates are high-energy compounds that are essential for various processes in the digestion, physiology, and metabolism of organisms, playing a crucial role in the growth and development of fish [33]. In slow-growing individuals, the liver is prompted to maintain normal life activities through lipid metabolism and gluconeogenesis, reducing the energy available for growth [34]. In this study, KEGG enrichment analysis revealed that pathways related to fat digestion and absorption and ether lipid metabolism were enriched; fat digestion and absorption and Type I diabetes mellitus were also enriched. In addition, GO terms such as organic substances metabolism process, macromolecule metabolism process, and organic cyclic compound binding were enriched. These biological processes have been reported to be related to the growth of *Pseudomonas* and *Oncorhynchus mykiss* [35,36]. In addition, as for DEGs related to lipid and carbohydrate metabolism, *apo B100*, *plin 1*, and *phyh* were highly expressed in the FG group, and *bsal* was highly expressed in the SG group. These genes are primarily involved in lipid transport, lipid synthesis, and cholesterol metabolism. The identification of a large number of lipid metabolism-related genes in this study further confirms their importance in fish growth. However, further study should be performed to determine their role in the growth of *S. sinensis*.

Additionally, fibronectin type III domain-containing 4 (*fndc4*), a component of type III fibronectin, and the transcription factor NF-κB were found to be highly expressed in the SG group. Previous studies have shown that FNDC4 and FNDC5 in humans can inhibit adipogenesis and induce adipocyte apoptosis, thus exerting anti-obesity effects [37], indicating the close relationship between *fndc4* and energy metabolism. Recent findings have demonstrated that FNDC4 can influence the differentiation of mouse myogenic cells and participate in muscle injury repair [38], providing evidence for its promoting role in skeletal muscle development. Furthermore, it was reported that there was a close correlation between the NF-κB pathway and *fndc4* expression [39], suggesting that NF-κB might play a similar role to fndc4 in the growth regulation of *S. sinensis*. Therefore, it is speculated that *fndc4* inhibits lipid synthesis, induces adipocyte apoptosis, and contributes to the lean phenotype of *S. sinensis*, while NF-κB may participate in skeletal growth and development and regulate myogenic differentiation similar to *fndc4*.

The alpha2-HS glycoprotein (*ahsg*) was identified to be up-regulated in FG and closely associated with insulin resistance [40]. Insulin is essential for tissue growth and development and the maintenance of systemic glucose homeostasis. It also affects lipid metabolism, increases lipid synthesis in adipocytes, and weakens fatty acids released by triglycerides in fat and muscle [41]. *ahsg* binds to the β subunit of insulin receptors, blocks insulin phosphorylation, and inhibits the insulin signaling pathway to regulate glucose and lipid metabolism [42]. Therefore, *ahsg* may promote the accumulation of fat and accelerate the growth of *S. sinensis* by regulating glycolipid metabolism.

In conclusion, this study conducted a comparative transcriptomic analysis of the hepatopancreas tissues of *S. sinensis* with different growth rates and results showed that the following mechanisms may be involved in the growth regulation of *S. sinensis*: (1) *sst2* as a gene on the neuroendocrine regulatory axis directly involved in regulating the growth of *S. sinensis*; (2) *fndc4* may cause *S. sinensis* to be lean by inhibiting lipid synthesis and inducing apoptosis of adipocytes; (3) *cckra*, a feeding-related gene, may play a regulatory role in the growth of *S. sinensis*. Based on the results of this study, further studies should be performed on the SNP-type polymorphism, which focuses on feeding regulation, to provide the basis for the selective breeding of fast-growth *S. sinensis.* Certainly, we cannot rule out whether these DEGs are affected by genetic differences and recommend that the fish used in future research should be from different families or fish whose genetic background is known and, if it is not possible to have them, to consider fish from the same parents (male and female).

## Figures and Tables

**Table 1:** Primers used for validation.

Gene ID	Annotation	Sequence
Cluster-30550.6193	Serine/Threonine-Protein Kinase 31 (*stk 31*)	F:AATTGCCAATGATCTATCC R:TCCTCCCACTCAACTCTT
Cluster-30550.62137	Probable G-Protein Coupled Receptor 149 (*gpr 149*)	F:GCATTCGCCACTGCCACTT R:GGTTCGCATCTCCGCTCCT
Cluster-35286.0	Angiopoietin-related protein 1 (*angptl 1*)	F: TGGGAATCACAGACGAGA R: GGAGAAACGAGGCACATA
Cluster-30550.68321	Follistatin-related protein 1 (*fstl 1*)	F: TGGCAGTAATGGGAAGACA R:CGGTCCGTAAGGTAACAAA
Cluster-30550.18115	Serine/Threonine-Protein Kinase SIK1 (*sik 1*)	F: TTCAGGAGGGAAGGAGAT R:ATACGGCTTTGTGGATTG
Cluster-30550.104720	Receptor Tyrosine Kinase Like Orphan Receptor 2 *(ror 2*)	F: ATCTGGCTGCTCGTAACATTCT R:TTGTAGTAGTCGGCTGAGTAGACC
Cluster-30550.59282	NLR Family CARD Domain Containing 3 (*nlrc 3*)	F: CACGCTCTGCCTGTTCTTA R: TCCATTTCCACGCTGTTAT
Cluster-30550.58994	PDZ And LIM Domain 2 (*pdlim 2*)	F:CGGACTCCAACTCCACCT R:GCACGGGATTCTTTGTTC
Cluster-30550.64357	Neuron Navigator 2 (*nav 2*)	F:GTTACCTGGCATCCTCTG R: TCGCTTCCTCCATTTACT
Cluster-30550.65732	Bone Morphogenetic Protein 1 (*bmp 1*)	F:AGAGGAGGCAGAGGACAG R: AAGAGCAGATTGCACCAG
Cluster-30550.15737	G-Patch Domain Containing 8 (*gpatch 8*)	F:CACCGATGATGAGATTGAGA R: GAGAAGAGGGCTGGAGTATG
Cluster-30550.7707	Somatostatin 2 (*sst 2*)	F:AAGGTCAGACAAGCCACA R:CCAAACAGAACAGGGAGA
Cluster-34620.0	Kruppel-Like Factor 6 (*klf 6*)	F:GGTGTTCGTGGGATGGCTGTG R:TGGCTGCATTTGAAAGGTTTGG
Cluster-36962.2	Cholecystokinin A Receptor (*cckar*)	F:TGCTGTAGGGATGATTTG R:CAGTTAGCCTTGTAAGTGTT
Cluster-30550.43338	Syncoilin, Intermediate Filament Protein (*sync 1*)	F: CCCAGATGTTTGAGCATAGCA R:TACCCTTCCTTCTTGGATTTA
Cluster-30550.53986	Basic Helix–Loop–Helix Family Member A15 (*bhlha 15*)	F:AGAGGCGTCTGTGAGGGTG R:AGGCGTTGTTGAGTTTGTG
Cluster-30550.15279	Stanniocalcin 1 (*stc 1*)	F:GAGCCATTCTCGACACTA R: CCCAAGAAGCACTTACAG
Cluster-34288.0	S100 Calcium-Binding Protein A1 (*s100a1*)	F: TGGTATTCCACCGTTATGC R: TCCTCAAAGTTCACCTCCC
Cluster-30550.58298	β-actin (*β-actin*)	F:TTCTTGGGTATGGAGTCTTG R:AGGTCCTTACGGATGTCG

**Table 2:** Statistics summary of RNA-seq data.

Sample	Raw Reads	Clean Reads	Clean Bases (%)	Error Rate	Q30 (%)	GC Content (%)
FG1	23,283,837	22,964,984	6.89	0.03	94.14	45.79
FG2	22,613,144	22,367,822	6.71	0.03	94.03	46.32
FG3	21,152,663	20,972,103	6.29	0.03	93.57	46.24
SG1	22,624,801	22,377,351	6.71	0.03	94.05	46.09
SG2	23,510,788	23,244,189	6.97	0.03	93.89	47.18
SG3	23,310,396	23,118,045	6.94	0.02	94.22	46.04
Assembly statistics (all the clean reads from the 6 libraries were assembled together)						
Term				Unigene		
N50				1635		
Total Length (bp)				157,542,467		
Max Length (bp)				51,530		
Min Length (bp)				301		
Average Length (bp)				1050		

## Data Availability

All the raw data obtained in this study have been submitted to the NCBI Sequence Read Archive (SRA) database under the project (PRJNA1109805).
